# The structural, electronic and optical properties of γ-glycine under pressure: a first principles study

**DOI:** 10.1039/c8ra08547a

**Published:** 2019-01-29

**Authors:** Aaron Mei, Xuan Luo

**Affiliations:** National Graphene Research and Development Center Springfield Virginia 22151 USA

## Abstract

The crystallized amino acid γ-glycine is a large band gap insulator that shows promise in the fields of photonics and non-linear optics. To better understand its physical properties, the effect of pressure on the structural, electronic and optical properties of γ-glycine were investigated through a first principles calculation approach based on density functional theory. A band gap of 5.026 eV was found and was shown to decrease with an increase of pressure, due to the widening of the conduction and valence bands. The densities of states verify the observations made in the band structure and exhibit that the conduction bands are dominated by the O 2p and C 2p orbitals. The absorption spectra for γ-glycine was calculated using many-body Green's functions GW and reveals a slight blueshift in the absorption spectra. The information presented in this paper might be useful in better understanding the structural and optical properties of γ-glycine for future application.

## Abstract 2

The crystallized amino acid γ-glycine is a large band gap insulator that shows promise in the fields of photonics and non-linear optics.

## Graphical abstract

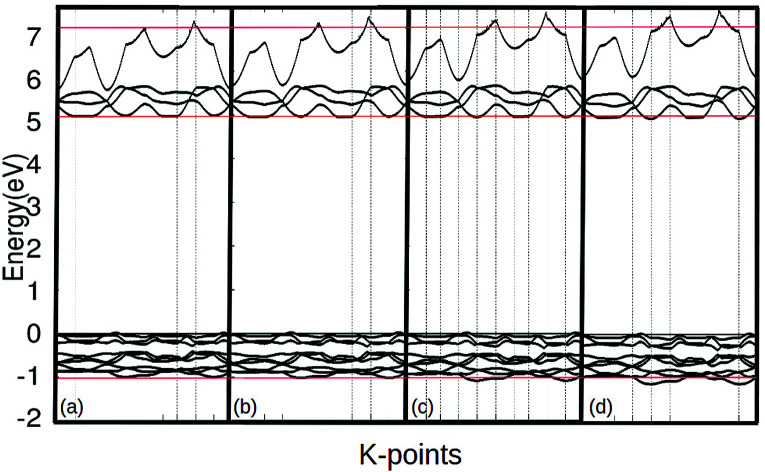

## I. Introduction

There are twenty different kinds of proteinogenic amino acids that can be found in the human genome. In nature, these amino acids are incorporated by organisms to synthesize proteins. However, it has recently been found that these amino acids possess the potential to be exploited and implemented in future technology with several attractive properties including thermal stability, mild piezoelectricity, and high nonlinear optical (NLO) coefficients.^1,15^ The simplest among these amino acids is glycine. Glycine is the only non chiral amino acid and crystallizes in three distinct polymorphs at ambient pressures known as α-glycine, β-glycine and γ-glycine. The most stable polymorph, γ-glycine crystallizes in the non-centrosymmetric space group of *P*3_1_ or *P*3_2_, making it ideal for piezoelectricity, NLO, and photonics.^3,19,21–23^

While there has been extensive research done on inorganic materials such as barium titanate, polyvinylidene fluoride, softer materials such as amino acids show more promise in areas such as regenerative medicine and energy harvesting, owing to their flexibility and low polarization.^9,11,16,29^ In addition to having a moderate piezoelectric response, γ-glycine also exhibits useful NLO properties as well, making it potentially useful in lasers, optical communications and data storage. In recent years, there have been many studies conducted on γ-glycine, with the majority of the research being experimental. The explored properties include band-gap, potential for second harmonic generation, reaction under various temperatures, and different methods of crystal growth.^2,7,8,21,25,35^ However, the results of the aforementioned experiments varied, as the observed band-gap and the absorption peaks range from 3.7 eV (Nithya 2015) to 6.49 eV (Azhagan 2017). Nonetheless, these experiments have revealed that γ-glycine holds potential as a valuable material in the field of optics with excellent NLO properties. Although many characteristics of γ-glycine have been explored, a theoretical study on its electronic structure and optical properties is still lacking.

It is well known that the optical properties of materials rely heavily on their electronic structures. High pressure is one method which can be used to regulate and control the electronic structure and optical properties of many materials.^4–6,12,24,26,27,30,31,36,37^ From previous observations, pressure has been shown to drastically alter the properties of amino acids, and can induce phase transitions.^13,32^ Thus, crystal structures that experience high pressures may result in sufficiently different, and potentially applicable changes in their optical and electronic properties.^17^ In this paper, we aim to investigate the effects of high pressure on the electronic structure and optical properties of γ-glycine. We hope that this work will provide some insight into the development of γ-glycine as an optical material.

In this study, first principle calculations were performed to analyze how the electronic and optical properties of γ-glycine reacts under various pressures. It has been established that γ-glycine undergoes a phase transition at around 2 GPa into ε-glycine, but does not complete it until 4.3 GPa.^12,33^ Consequently so, simulations of high pressures up to 3.0 GPa at intervals of 1.0 GPa were created and the electronic band structure, absorption spectra were then calculated using the structural results. In Section II, we detailed our methods to perform the calculations for γ-glycine under high pressures. In Sec. III, we presented our results on how the high pressures affected the electronic band structure and absorption spectra of γ-glycine, and compared our results with experimental and other theoretical research. Finally, our conclusion can be found in Sec. IV.

## II. Computational methods

Using the first-principles calculation method based on density functional theory (DFT) as implemented in ABINIT,^14^ the electronic and optical properties of γ-glycine under different pressures were investigated with the Generalized Gradient Approximation (GGA)^28^ and the Perdew–Burke–Ernzerhof (PBE) exchange correlation function. To describe the interaction between ions and electrons, the Projected Augmented Wave (PAW) method^10,34^ was used with projectors generated with the ATOM code^18^ and the pseudopotentials of the elements were generated with the electron configurations and radius cutoffs of each element. The electron configurations of the elements were 1s^1^ with a radius cutoff of 0.995 Bohr, [He]2s^2^2p^2^with a radius cutoff of 1.507 Bohr, [He]2s^2^2p^3^ with a radius cutoff of 1.200 bohr and [He]2s^2^2p^4^ with a radius cutoff of 1.415 bohr for hydrogen, carbon, nitrogen and oxygen respectively.

To balance computational time and accuracy, the computational parameters were converged. The tolerance for the self consistent field cycles was set to 1.0 × 10^−10^ Hartree (Ha), and a dataset was declared converged when the differences in total energies for the following two datasets pertained to less than 1.0 × 10^4^ Ha per cell. The kinetic energy cutoff was converged individually for each element, (H, C, N, O) and the greatest cutoff was used. The structural optimization of the atoms was conducted by applying the Broyden–Fletcher–Goldfarb–Shanno minimization at 0 K with a maximal force tolerance of 5.0 × 10^−4^ Ha per bohr.

In the investigation of the effect of pressure on the electronic structure and optical properties of γ-glycine, discrete hydrostatic pressures (0.0, 1.0, 2.0, 3.0) were applied. The lattice parameters were changed to achieve a new volume based off of target stresses tensors by re-relaxing the structure^19,20^ To reduce the effect of Pulay stress, the energy cutoffs were increased by 20% during relaxation.

**Fig. 1:**
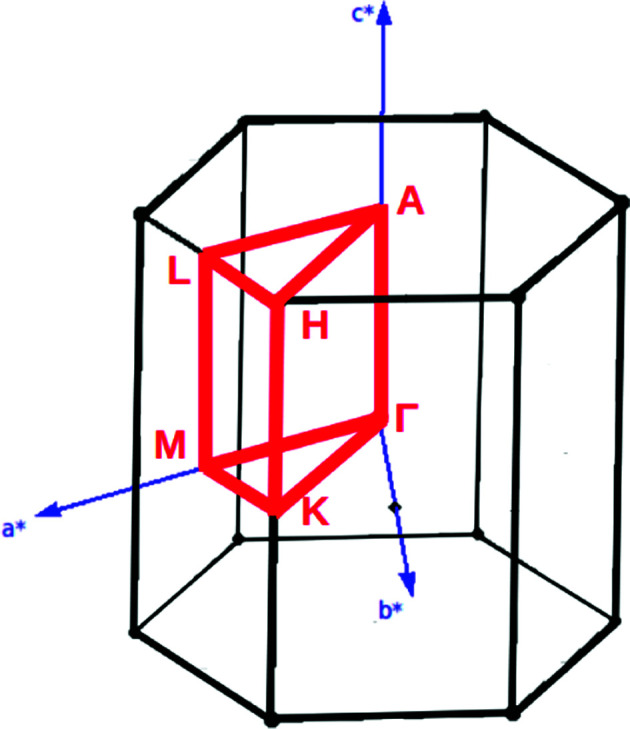
First Brillouin zone of hexagonal space group.

Analysis and further calculations were then performed using the pressure modified crystal structures. The unit cells for glycine were calculated in reciprocal space according to eqn (1)–(3). High symmetry *k*-points were then chosen to comprehensively sample the first Brillouin zone (Fig. 1) to construct the band structure.1
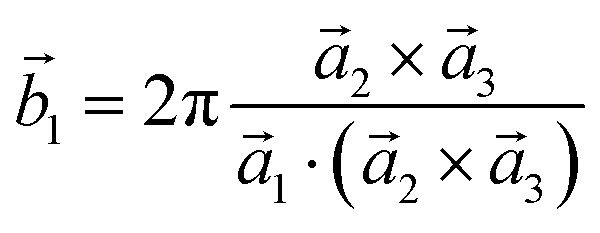
2
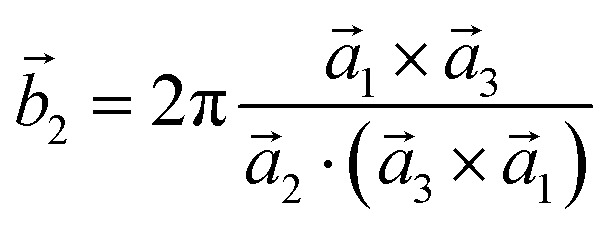
3
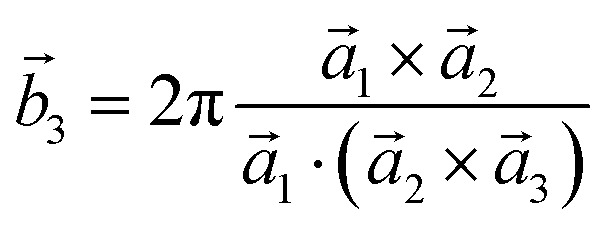


Many body perturbation theory was applied along with the GW method to calculate the absorption spectra for γ-glycine. Before calculating the absorption, the number of bands in transition space, and the energy cutoff for the dielectric matrix were converged. To avoid divergencies during the evaluation of the Adler–Wiser expression of the irreducible polarizability, a complex shift of 0.10 was used. For the number of bands and the energy cutoff for the dielectric matrix, the values were considered converged when the energy between the valence band and conduction band were less than 0.01 eV during perturbation.

## III. Results and discussion

First principle calculations were used to investigate the electronic and optical properties of γ-glycine. The greatest kinetic energy cutoff among the elements converged at 25 Ha from Nitrogen and was therefore used for further calculations. The convergence study on the *k*-point mesh resulted in a grid of 4 × 4 × 4 with an applied shift of 0.0 0.0 0.5 to maintain symmetry. Before applying pressure to the crystal, the structure was relaxed until the forces and energy. A series of different hydrostatic pressures were then applied according to the approximate stress tensors inside of ABINIT on the basis of the relaxed atomic structure.

### A. Structural determination

**Fig. 2:**
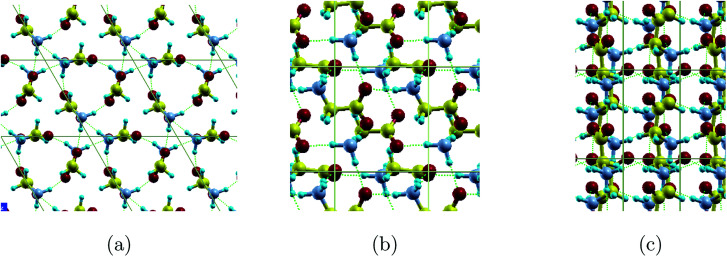
Blue: hydrogen yellow: carbon grey: nitrogen red: oxygen (a) crystal structure of glycine depicted along the *z* axis (b) crystal structure of glycine depicted along the *x* axis (c) crystal structure of glycine depicted along the *y* axis.

**Fig. 3:**
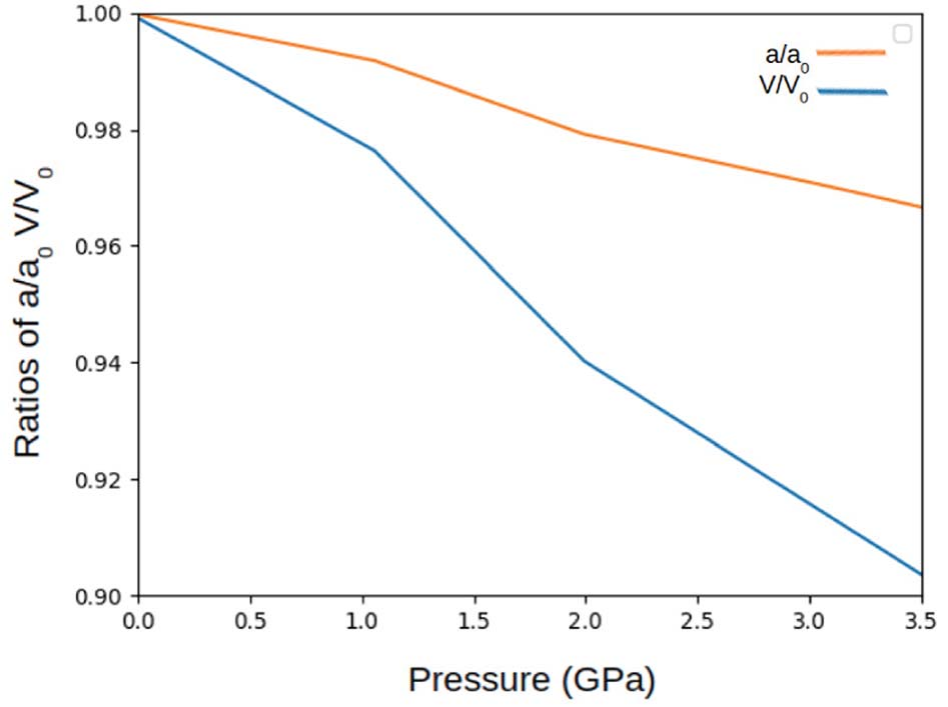
Change in the lattice constant and volume ratios under pressure.

**Fig. 4:**
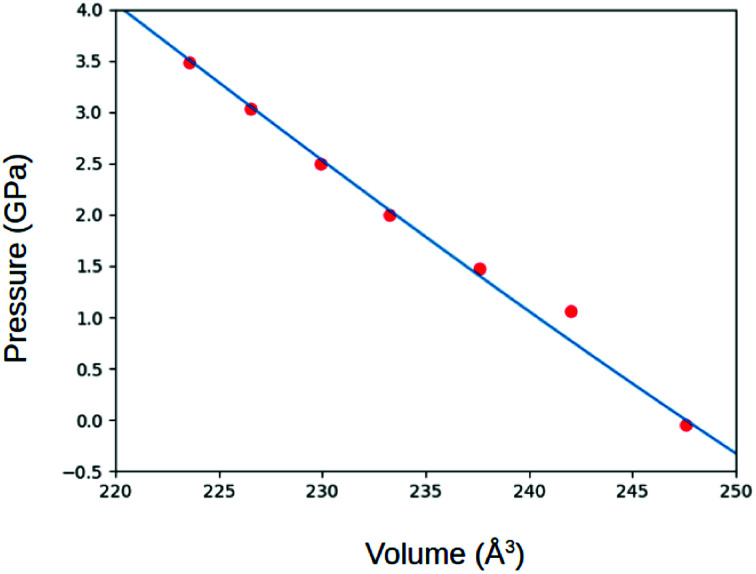
The pressure applied on the cell as a function of the volume.

In preparation of further calculations and analysis, the geometrical structure of γ-glycine was optimized. (Fig. 2). The space group of *P*3_1_ was used with the initial lattice parameters being *a* = 7.037 Å *c* = 7.037 Å from Iitaka. The lattice parameters after geometric relaxation were found to be *a* = 7.159 Å *c* = 5.58 Å with a deviation of 1.73%. The discrepancy in lattice parameters may be due to that the relaxation was performed at 0 K while the experimental data was recorded at room temperature. Upon further observation, it was found that as the pressure on the cell increased, the lengths of the lattice cell decreased along with the overall volume of the unit cell (Fig. 3 and 4). This decrease in the volume results in a disturbance of the interaction between the atoms, making the electronic structure and optical properties of γ-glycine subject to change.

### B. Electronic structure

**Fig. 5:**
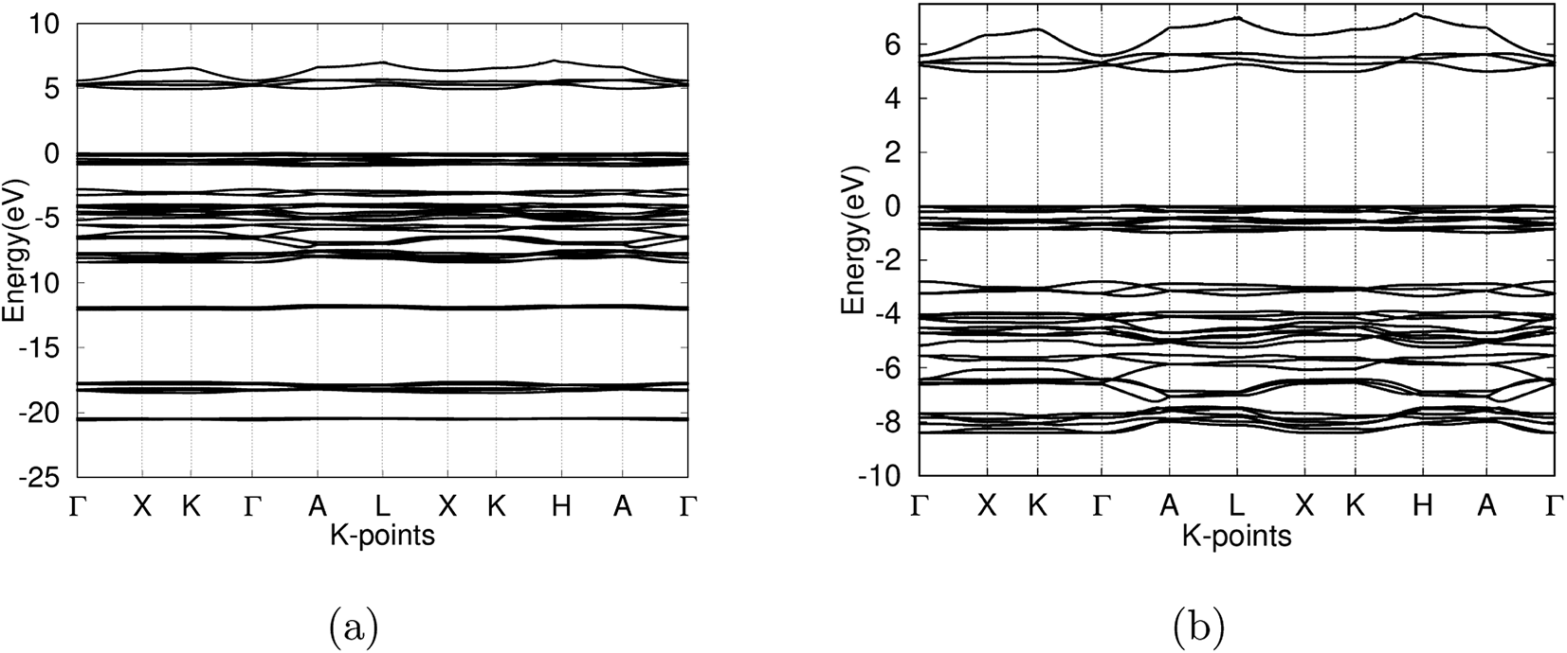
(a) Band structure of γ-glycine crystal in the −25 to 10 eV calculated with the PAW method (b) detail of the band structure of γ-glycine near the Fermi level (−10 eV to 7.5 eV).

After the optimization of the γ-glycine crystal at various pressures, the electronic band structure and the density of states were constructed. At ambient pressures, the fundamental band gap was calculated to be 5.005 eV (Fig. 5) and the optical band gap was 5.026 eV with a Fermi level of 0.295 eV, which falls in the range of previous experimental results.^2,7,8,25,35^ The large band gap calculated confirms γ-glycine as an insulator, as well as its viability as a candidate for NLO applications.

**Fig. 6:**
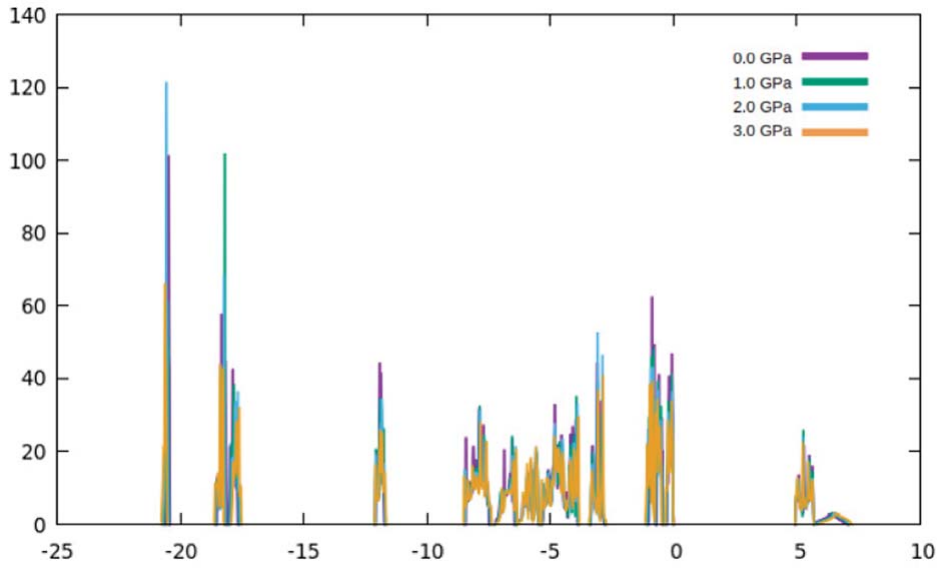
The densities of states under different pressures.

The valence band of γ-glycine consists of five parts, a high energy region near the Fermi level, two medium regions (−8.5 eV to −2 eV and near −12 eV), and two low energy regions (near −18 eV and −21 eV), while there is only one region in the conduction band. From Fig. 5, it can be observed that the band structure spans between −21 eV and 7.5 eV with most of the bonding occurring between the −8.5 eV and the Fermi level. The atomic contributions to the densities of states are shown in Fig. 6. The energy bands near the −20 eV originate from the O 2s and C 2s orbitals, the bands close to the −17.5 eV energy region are dominated by the N 2s and O 2s orbitals, and the bands around −12.5 eV are from the C 2s orbitals. Most of the bands reside in the region between −8.5 eV and −2.5 eV, and have major contribution from the H 1s, N 2p, C 2p and O 2p orbitals, and the upper valence bands between −1 and 0 eV result from the O 2p orbitals with a slight contribution from the N 2p orbitals. The conduction bands are mainly dominated by the C 2p orbitals with contributions from the O 2p orbital as well.

As the pressure applied to the cell increases, a slight decrease in the band gap is experienced. This is because the application of pressure results in a reduction in bond lengths, which causes an increase in interaction and orbital overlap. The resulting increase in orbital overlap allows for easier electron hopping and therefore decreases the band gap. With the increased pressure, the overlapped bands seen in Fig. 9, start to spread apart slightly as the pressure in the system increases the band structure expands. In Fig. 9, it can be seen with the application of pressure, the conduction and valence bands both start to widen, causing a decrease in the band gap.

**Fig. 7:**
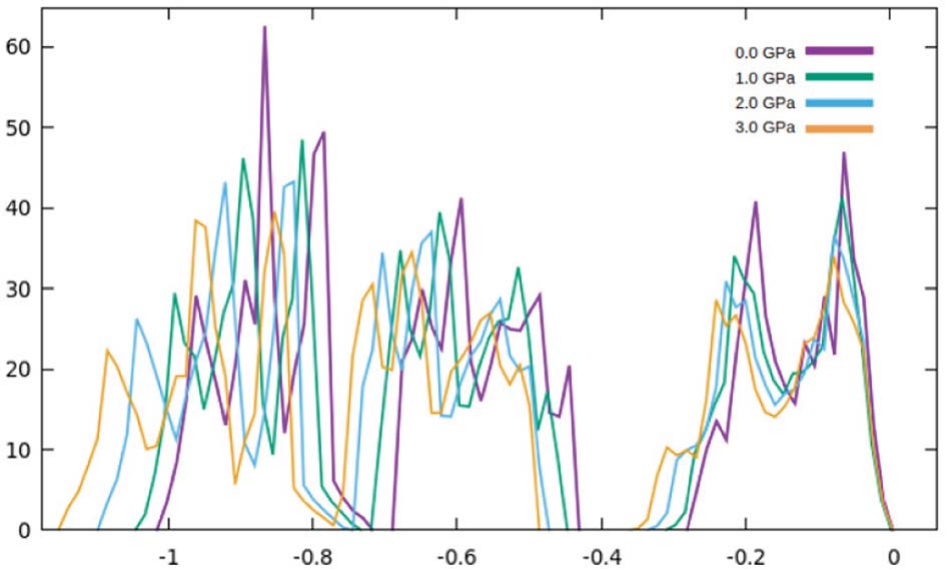
The densities of state at a short energy range from −1.2 eV to 0.0 eV.

**Fig. 8:**
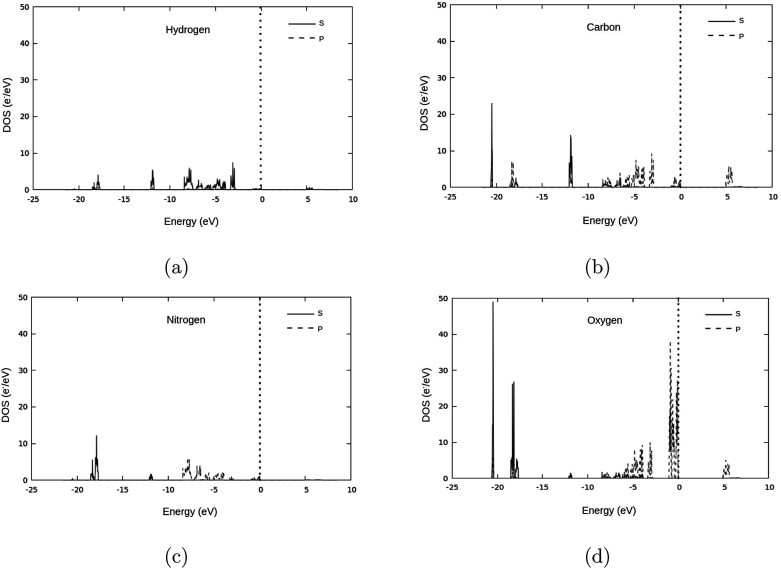
Atomic group contributions to the density of states of γ-glycine.

In addition to modifications to the band gap, the increase of interaction also influences the overall energy of the cell, with the Fermi level rising 0.13 eV per GPa. However, further investigating the DOS, (Fig. 7 and 8) it can be seen that the shapes of the peaks only deform slightly under pressure, suggesting that no phase transitions occurred up to 3.0 GPa.

### C. Optical properties

It is key to understand the absorption spectrum of γ-glycine under pressure considering its inherent potential in the fields of non-linear optics and photonics. When light is absorbed, the electrons inside of the crystal become excited by the incoming photons and can transit from the valence bands to the conduction bands given a sufficient amount of energy. Therefore, the band gap is directly analogous with the optical properties of γ-glycine and a change in the band gap is likely to induce a change in the absorption spectrum as well.

The optical properties of γ-glycine are calculated through the frequency-dependent dielectric function, given by *ε*(*ω*) = *ε*_1_(*ω*) + *iε*_2_(*ω*) the imaginary part, given by *ε*_2_(*ω*) represents the absorption and can be directly calculated by using4
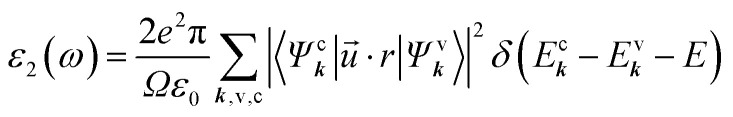
where *ω* is the frequency of the incoming light, and *Ψ*^c^_***k***_ and *Ψ*^v^_***k***_ represent the conduction and valence band wave functions at ***k***, respectively. The vector *u⃑* defines the polarization of the incident electric field. The real part, *ε*_1_ can then be derived from *ε*_2_ using the Kramers–Kronig transform. The real and imaginary parts of the refraction index, *n* and *k*, can then be found through

5 *ε*_1_ = *n*^2^ − *k*^2^

6 *ε*_2_ = 2*nk*

Using this information, the optical absorption of a crystal can then be calculated with7
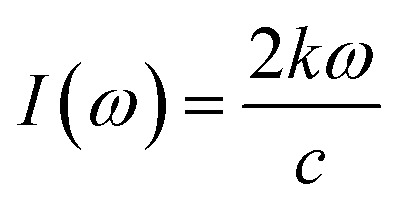


In Fig. 10, the optical absorption for γ-glycine is presented with a range from 1.0 eV to 6 eV calculated with the GGA functional. In the calculated photon range, the absorption spectrum has a clear single absorption peak at 5.95 eV using GW.

**Fig. 9:**
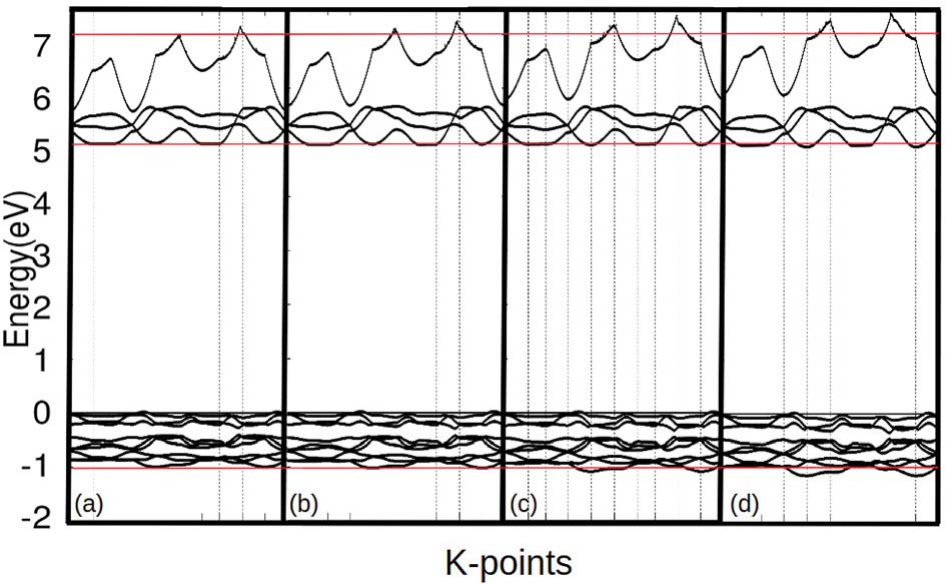
The band structure of γ-glycine crystal under pressures of 0 (a), 1.0 (b), 2.0 (c), 3.0 (d) GPa near the Fermi level.

**Fig. 10:**
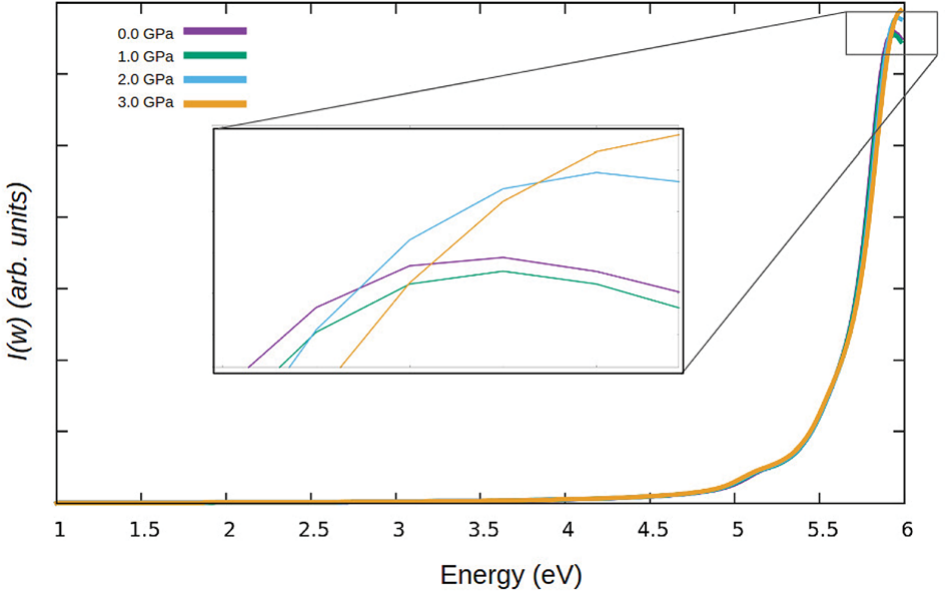
The absorption spectrum for γ-glycine calculated using GW.

The calculated absorption spectrum is in good agreement with previous theoretical and experimental values in terms of relative peak height and positioning. The absorption spectrum falls within the deep UV region of the electromagnetic spectrum. It can be observed that the graph displays low absorption from 300–1200 nm, confirming that γ-glycine is transparent in the visible region. Furthermore, the absence of absorption in the region between 250 and 1200 nm is crucial for non linear optical devices, therefore making γ-glycine a viable candidate for non linear optical applications. Although the band gap narrows, the absorption spectrum does not redshift. Instead, a slight, consistent blueshift the UV region as shown in Fig. 10, with the absorption beaks variably contracting and expanding. The blueshift in the absorption spectrum of γ-glycine under pressure as well as the fluctuations in the absorption peaks, may be attributed to the phase transition to the polymorph ε-glycine under pressure, as during this phase transition, defects may occur in the crystal structure which can cause variations in the absorption spectrum.

## IV. Conclusion

In this paper, the structural, electronic and optical properties of γ-glycine were investigated under various pressures through first principles DFT-GGA calculations. The obtained unit cell volume from structural relaxation at ambient pressures was slightly larger but in good agreement previous experimental results. As the pressure of the system increases, the normalized structural parameters decrease. The study of the densities of states with respect to pressure revealed that DFT shows that there are no drastic structural changes in the pressure range of 0.0–3.0 GPa, and therefore establishes that γ-glycine displays electronic stability in this region. The large band gap obtained demonstrates that γ-glycine is an insulator and is therefore feasible as a material for NLO applications, which is further affirmed by the absorption spectra. The absorption spectra obtained from GW resulted in a peak in the range of deep UV and presented a blueshift with an increase of pressure. Non-linear devices and photonics are set to be prevalent in our everyday lives with future applications in quantum optics, quantum computing, plasma physics, and laser manufacturing. It has been consistently shown that the prospective material -glycine is a crystallized amino acid which can be utilized in these fields. The predictions presented in this work might provide new insight into the application of -glycine in future technology.

## Conflicts of interest

There are no conflicts to declare.

## Supplementary Material
